# HbA_1c_ as a Screening tool for Ketosis in Patients with Type 2 Diabetes Mellitus

**DOI:** 10.1038/srep39687

**Published:** 2016-12-23

**Authors:** Bing Zhu, Le Bu, Manna Zhang, Aaron M. Gusdon, Liang Zheng, Sharvan Rampersad, Jue Li, Shen Qu

**Affiliations:** 1 Department of Endocrinology and Metabolism, Shanghai Tenth People’s Hospital, School of Medicine, Tongji University, Shanghai, China; 2 Department of Neurology and Neuroscience, Weill Cornell Medical College, New York-Presbyterian Hospital, New York, NY, United States of America; 3 Heart, Lung and Blood Vessel Center, Tongji University, Shanghai, China; 4 Department of Endocrinology, School of Medicine, Nanjing Medical University, Nanjing, China

## Abstract

Ketosis in patients with type 2 diabetes mellitus (T2DM) is overlooked due to atypical symptoms. The objective of this study is to evaluate the value of hemoglobin A_1c_ (HbA_1c_) as a screening tool for ketosis in T2DM patients. This retrospective study consisted of 253 T2DM patients with ketosis at Shanghai 10th People’s Hospital during a period from January 1, 2011 to June 30, 2015. A control group consisted of 221 T2DM patients without ketosis randomly selected from inpatients during the same period. Receiver operating characteristic curve (ROC) analysis was used to examine the sensitivity and specificity of HbA_1c_ as an indicator for ketosis. Higher HbA_1c_ levels were correlated with ketosis. In patients with newly diagnosed T2DM, the area under the curve (AUC) was 0.832, with 95% confidence interval (CI) 0.754–0.911. The optimal threshold was 10.1% (87 mmol/mol). In patients with previously diagnosed T2DM, the AUC was 0.811 (95% CI: 0.767–0.856), with an optimal threshold of 8.6% (70 mmol/mol). HbA_1c_ is a potential screening tool for ketosis in patients with T2DM. Ketosis is much more likely with HbA_1c_ values at ≥10.1% in patients with newly diagnosed T2DM and HbA_1c_ values at ≥8.6% in patients with previously diagnosed T2DM.

Ketosis-prone type 2 diabetes is defined as the A-β+ ketosis-prone diabetes (KPD) subgroup^1^. This subgroup is a major factor driving the increasing prevalence of KPD^2^,^3^,^4^,^5^,^6^,^7^. The term “ketosis-prone type 2 diabetes (T2DM)” is often used to describe the A-β+ patients who present with new onset diabetes, unprovoked diabetic ketoacidosis (DKA)^8^,^9^ and acidosis^10^,^11^,^12^. As a result, the prevalence of ketosis-prone T2DM could be grossly underestimated. In comparison with DKA in type 1 diabetes mellitus (T1DM), DKA in T2DM is more intractable^7^,^13^. DKA in T2DM patients is more likely to develop into severe forms^13^ and also requires higher doses of insulin and longer durations of treatment^7^. T2DM patients with ketosis but no acidosis often do not present with overt clinical symptoms. As such, failure to recognize ketosis also likely contributes to the worse outcomes^7^.

HbA_1c_ reflects average blood glucose over the past 2–3 months^14^. Several reports have indicated the utility of HbA_1c_ in predicting the development of diabetic retinopathy and nephropathy^15^,^16^,^17^,^18^. The mean HbA_1c_ is reported to be higher than 10% in T2DM patients with ketosis^9^,^19^,^20^,^21^. Considering the fact that ketosis is the end result of prolonged uncontrolled diabetes^22^,^23^, we hypothesized that HbA_1c_ could be used as a screening tool for ketosis in T2DM patients.

## Results

### Patient characteristics

In comparison to the control subjects, the ketosis group had a higher percentage of males (66.8% vs. 63.8%, *P* = 0.494; Table 1) and was younger (50.9 ± 18.1 vs. 55.0 ± 16.6, *P* = 0.01). Patients with ketosis also had higher HbA_1c_ (11.5% ± 2.4% vs. 8.5% ± 2.0%, *P* < 0.001), higher fasting plasma glucose (FPG) and 2h-postprandial plasma glucose (PG) levels (*P* < 0.001), lower fasting C-peptide levels (*P* < 0.001), and lower 2h-postprandial insulin and C-peptide levels (*P* < 0.001). No significant differences were found in body mass index (BMI), systolic blood pressure (SBP), diastolic blood pressure (DBP), heart rate (HR), hemoglobin (Hb), arterial pH, bicarbonate, osmolality, fasting insulin, serum creatinine (sCr), blood urine nitrogen (BUN), uric acid (UA), glutamic-pyruvic transaminase (ALT), glutamic-oxalacetic transaminase (AST), low density lipoprotein (LDL), and high density lipoprotein (HDL) levels between the two groups, with the exception of cholesterol (TC) (4.9 ± 1.5 vs. 4.6 ± 1.1, *P* = 0.006), triglycerides (TG) (1.4 (1.0, 2.5) vs. 1.4 (1.0, 2.1), *P* = 0.016), and free fatty acid (FFA) levels (0.6 ± 0.3 vs. 0.5 ± 0.2, *P* < 0.001). Among patients with ketosis, subjects with a known history of T2DM had lower HbA_1c_ than in subjects with newly diagnosed T2DM (12.3 ± 2.0 vs. 11.1 ± 2.5, *P* < 0.001; Supplemental Table S1).

### Relationship between HbA_1c_ and ketosis

**Figure 1:**
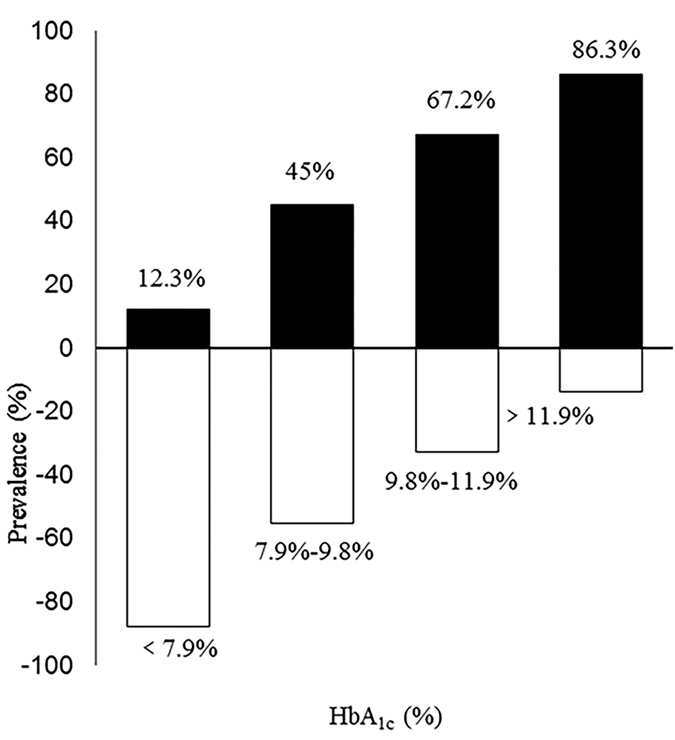
The prevalence of type 2 diabetic ketosis with increasing levels of HbA_1c_. HbA_1c_ was plotted in quartiles with the HbA_1c_ levels set at ≤7.9%, 7.9–9.8%, 9.8–11.9%, and ≥11.9%. Black bars = proportions of patients with type 2 diabetic ketosis. White bars = proportions of type 2 diabetes patients without ketosis.

Higher HbA_1c_ was positively correlated with urine ketones (*r* = 0.54, *P* < 0.001) as well as plasma ketones (*r* = 0.58, *P* < 0.001). HbA_1c_ was plotted in quartiles with the HbA_1c_ levels set at <7.9%, 7.9–9.8%, 9.8–11.9%, and ≥11.9%. As expected, the occurrence of ketosis increased rapidly with increasing levels of HbA_1c_ (12.3%, 45.0%, 67.2% and 86.3%, per HbA_1c_ quartile respectively) and exhibited a sevenfold increase from the lowest to the highest quartile (Fig. 1). In the multivariate model 1 that included age, gender and C-reactive protein (CRP) as co-variables, HbA_1c_ was significantly associated with ketosis (odds ratio (*OR*) = 1.87, 95% confidence interval (CI) 1.64 to 2.13, *P* < 0.001; Table 2). In the multivariate model 2 with BMI, smoking, drinking, and duration of diabetes as additional co-variables, the association between HbA_1c_ and ketosis remained (*OR* = 1.88, 95% CI 1.64 to 2.15, *P* < 0.001; Table 2).

### Determination of optimal HbA_1c_ thresholds

**Figure 2:**
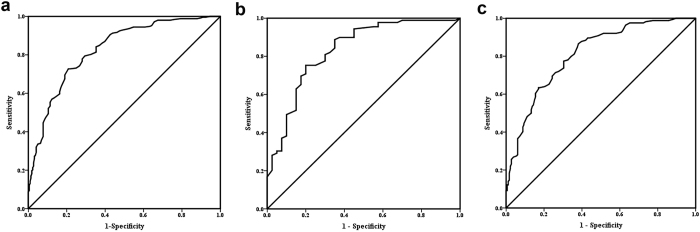
Receiver operating characteristics curve of HbA1c in screening for diabetic ketosis in type 2 diabetes patients. (**a**) Total group: area under curve (AUC) were 0.827 (95% confidence interval (CI) 0.791 to 0.864). (**b**) The subgroup of patients with newly diagnosed type 2 diabetes: AUC were 0.832 (95% CI 0.754 to 0.911), cut-off point  = 10.1%, sensitivity  = 88.76%, specificity  = 65.00%. (**c**) The subgroup of patients with previously diagnosed of type 2 diabetes: AUC were 0.811 (95% CI 0.767 to 0.856), cut-off point  = 8.6%, sensitivity  = 86.59%, specificity  = 62.00%.

In the receiver operating characteristics (ROC) analysis, area under the curve (AUC) was 0.827 (95% CI: 0.791–0.864) for the overall analysis that included all subjects, 0.832 (95% CI: 0.754 to 0.911) in patients with newly diagnosed T2DM, and 0.811 (95% CI: 0.767 to 0.856) in patients with a known T2DM history (Fig. 2). In patients with a known T2DM history, a HbA_1c_ threshold of 8.6% (70 mmol/mol) resulted in the highest Youden index, with 86.59% sensitivity, 62.00% specificity, and 0.22 negative likelihood ratio (LR) (Table 3). In patients with newly diagnosed T2DM, a HbA_1c_ threshold of 11.0% (97 mmol/mol) resulted in the highest Youden index, with 75.30% sensitivity and 80.00% specificity. A HbA_1c_ threshold of 10.1% (87 mmol/mol) seemed optimal with the second highest Youden index, with 88.76% sensitivity, 65.00% specificity, and 0.17 negative LR (Table 4). In subjects with a known T2DM diagnosis, the adjusted OR for having ketosis in individuals with HbA_1c_ levels greater than or equal to 8.6% (70 mmol/mol) vs. lower than 8.6% was 12.49 (95% CI: 6.35 to 24.56) (Supplemental Table S2). In subjects with newly diagnosed T2DM, the adjusted OR (95% CI) for having ketosis in individuals with HbA_1c_ levels greater than or equal to 10.1% (87 mmol/mol) vs. lower than 10.1% was 27.58 (95% CI: 7.77 to 97.88) (Supplemental Table S2).

### Oral glucose tolerance tests (OGTTs) analysis

**Figure 3:**
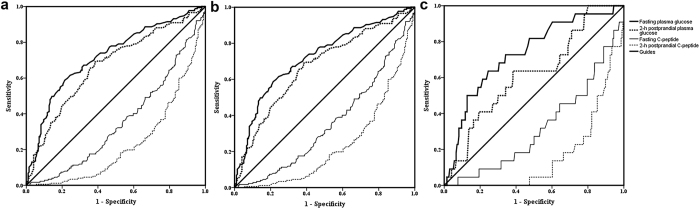
Receiver operating characteristics curve of oral glucose tolerance tests (OGTTs) in screening for type 2 diabetic ketosis. (**a**) Total group: area under curve (AUC) were 0.712 (95% CI 0.664 to 0.760) for fasting plasma glucose (FPG), 0.666 (0.613 to 0.720) for 2-h postprandial plasma glucose (PG), 0.337 (0.287 to 0.388) for fasting C-peptide and 0.243 (0.196 to 0.290) for 2-h postprandial C-peptide. (**b**) The subset of patients with diabetic ketosis without acidosis: AUC were 0.771 (0.661 to 0.762) for FPG, 0.672 (0.616 to 0.727) for 2-h postprandial PG, 0.354 (0.301 to 0.407) for fasting C-peptide and 0.252 (0.202 to 0.301) for 2-h postprandial C-peptide. (**c**) The subset of patients with diabetic ketoacidosis: 0.717 (0.623 to 0.810) for FPG, 0.631 (0.514 to 0.747) for 2-h postprandial PG, 0.241 (0.152 to 0.330) for fasting C-peptide and 0.184 (0.112 to 0.255) for 2-h postprandial C-peptide.

In the overall analysis that included subjects with ketosis regardless of having acidosis or not, the AUC was 0.712 (95% CI: 0.664 to 0.760) for FPG, 0.666 (0.613 to 0.720) for 2-h postprandial PG, 0.337 (0.287 to 0.388) for fasting C-peptide, and 0.243 (0.196 to 0.290) for 2-h postprandial C-peptide (Fig. 3a). In the subset with ketosis but not acidosis, the AUC was 0.771 (0.661 to 0.762) for FPG, 0.672 (0.616 to 0.727) for 2-h postprandial PG, 0.354 (0.301 to 0.407) for fasting C-peptide, and 0.252 (0.202 to 0.301) for 2-h postprandial C-peptide in the subset of patients with ketosis without acidosis (Fig. 3b). In the subset with ketoacidosis, the AUC was 0.717 (0.623 to 0.810) for FPG, 0.631 (0.514 to 0.747) for 2-h postprandial PG, 0.241 (0.152 to 0.330) for fasting C-peptide, and 0.184 (0.112 to 0.255) for 2-h postprandial C-peptide (Fig. 3c).

## Discussion

In this study we found a significant association between higher HbA_1c_ values with ketosis in T2DM patients. The optimal threshold for screening ketosis was 10.1% (87 mmol/mol) and 8.6% (70 mmol/mol) in patients with newly diagnosed T2DM and in patients with a known T2DM history, respectively. These results provide a pragmatic tool to screen for ketosis in patients with T2DM.

The mean HbA_1c_ of T2DM patients reported in this study is similar to that reported in previous studies^9^,^19^,^20^,^21^. DKA was demonstrated to be associated with increased HbA_1c_ levels which reflect both fasting and postprandial hyperglycemia^24^ in T1DM^23^,^25^,^26^,^27^ and T2DM^28^. Our results provide further evidence to support the relevance of HbA_1c_ levels and risk of ketosis in T2DM. In addition, Cheng PC *et al*. have demonstrated that serum albumin concentration, which is inversely associated with HbA_1c_^29^,^30^,^31^, is inversely associated with the risk of ketosis in patients with T2DM^29^. However, few research studies have concentrated on the value of HbA_1c_ as a screening tool for ketosis in T2DM. The pathogenesis of ketosis likely involves decreasing effective concentrations of insulin as well as increased concentrations of glucagon, cortisol, growth hormone and catecholamines, which promote lipolysis and ketogenesis^32^,^33^ and trigger ketonemia and DKA. Moreover, insulin deficiency and increased counterregulatory hormones inhibit glucose utilization in peripheral tissues, promoting gluconeogenesis and glycogenolysis, thereby exacerbating hyperglycemia^34^. In addition, there are some interactions between hyperglycemia and disturbances in lipid metabolism. We found that patients with diabetic ketosis had high plasma FFA, TC, and TG levels. Excess FFA in the liver stimulate gluconeogenesis^35^. Thus, dyslipidemia and disturbances in glucose metabolism can be distinct consequences of the same cause. Hyperglycemia coexists with ketosis rather than as a cause of it. In our opinion, measures of glucose metabolism could reflect lipid metabolism to some degree. This is in line with the America Diabetes Association (ADA)’s recommendation that plasma glucose is a key diagnostic criteria for DKA^34^. Previous studies also have indicated that HbA_1c_ can provide valuable supplementary information about the extent of circulating lipids in both T1DM and T2DM in addition to its primary role in monitoring long-term glycemic control^36^,^37^,^38^,^39^,^40^. The observed correlation of HbA_1c_ with ketosis in the current study provides additional evidence that links HbA_1c_ with disturbances in lipid metabolism in T2DM patients.

The Youden index in ROC analysis is commonly used to measure overall diagnostic performance^41^,^42^. In the subgroup of patients with a known T2DM history, the cutoff value with the highest Youden index was 8.6% (70 mmol/mol), with a high sensitivity (86.59%) and moderate specificity (62.00%). The low negative LR (0.217) indicates good discriminatory performance and a lower rate of false negatives^43^. In the subgroup of patients with newly diagnosed T2DM, the highest Youden index was obtained at a cutoff of 11.0%, with 75.30% sensitivity and 80.00% specificity. Considering the severe adverse outcomes of diabetic ketoacidosis, such as death, and the heavy economic burden of hospitalization^34^, we placed particular emphasis on sensitivity in the current study, and set the HbA_1c_ threshold at 10.1% (87 mmol/mol; with second highest Youden index); at this cutoff, the analysis yielded higher sensitivity (88.76%), moderate specificity (65.00%), and low negative LR (0.173). Distinct optimal HbA_1c_ thresholds between the two subgroups may relate to fewer changes in the counter regulatory hormone system in patients with newly diagnosed T2DM than in those with long standing diabetes^44^. Furthermore, almost all patients with a known diagnosis of T2DM had initiated medications for diabetes, which may additionally influence the optimal threshold value.

It is note worthy that HbA_1c_ showed better performance than OGTTs in the current study. In addition, OGTTs require that patients fast for at least 8 hours before examination, and short term dietary control or physical exercise can influence the results. This test is also an expensive and lengthy procedure that requires additional manpower and professional expertise. Moreover, there are stringent requirements for processing blood during OGTTs including rapid processing as well as separation and storage of plasma or serum at 4 °C^18^. In contrast, HbA_1c_ levels can be checked at any time of a day without fasting and accurately reflect long term glycemic control without susceptibility to short term changes in diet or exercise. In addition to be more cost-effective, HbA_1c_ is more reproducible than OGTTs^45^. Also, blood samples for HbA_1c_ measurement can be maintained at 4 °C for up to a week^18^. Importantly, instant blood or urine ketone measurements can determine those with ketosis but are unable to recognize those at high risk of developing ketosis. In contrast, the ability of HbA_1c_ to reflect glycometabolic status over several months may allow identification of patients who are at high risk for developing ketosis.

Although this study has addressed some knowledge gaps in the use of HbA_1c_ to screen for ketosis, there are several limitations. Most importantly, this is a retrospective case-control study, which does not provide evidence as strong as randomized controlled trials. Furthermore, the design of this study could have generated selection bias: all subjects were from one hospital and of a single ethnic background (Chinese Han), which limits the generalizability of the study findings. Also, all subjects in the current study were inpatients; as a result, whether the findings can be extrapolated to outpatients needs to be verified. Due to less severity of diseases in the outpatient setting, we believe that a lower HbA_1c_ threshold may appropriate in outpatients. Finally, confounding factors such as the various comorbidities among the ketosis and control group patients may weaken the study findings.

In conclusion, HbA_1c_ is a useful tool to screen T2DM patients at high-risk for ketosis. We believe that plasma and urine ketones should be monitored carefully while appropriate treatments should be implemented in patients with newly diagnosed T2DM with HbA_1c_ at ≥10.1% (87 mmol/mol) and in patients with a known T2DM history having a HbA_1c_ value at ≥8.6% (70 mmol/mol) at the time of admission.

## Materials and Methods

### Ethics statement

The study protocol was approved by the Research Ethics Review Committee of Tongji University. Methods used in the present study were carried out in accordance with approved guidelines and regulations. It conformed to the provisions of the Declaration of Helsinki.

### Study population

**Figure 4:**
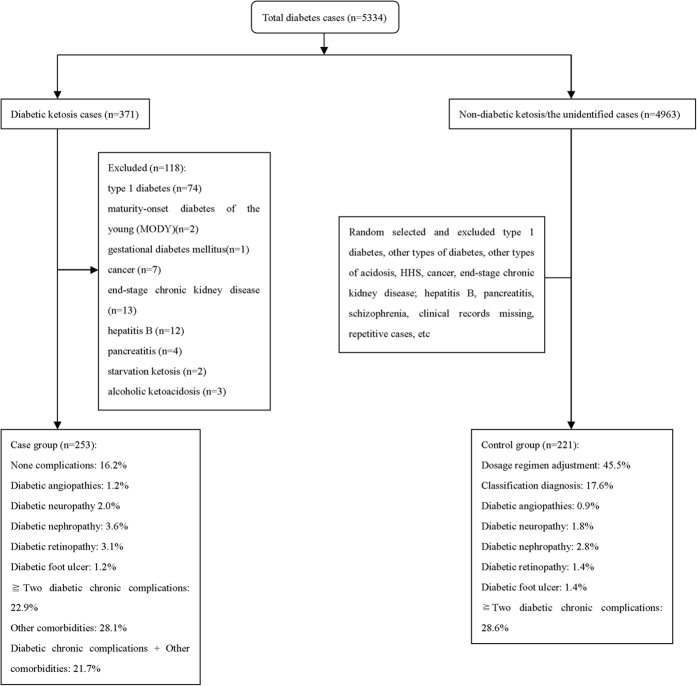
Diagram demonstrating the case and control selection and exclusion criteria used in this study.

This retrospective case–control study was conducted at the Department of Endocrinology and Metabolism, Shanghai 10th People’s hospital in China, a 1,860-bed comprehensive teaching hospital, from January 1, 2011 to June 30, 2015. In the initial screening step of the study, we identified a total of 5,334 T2DM patients receiving medical treatment as inpatients and they did not undergo any surgical procedure. Ketosis without anaemia was verified in 371 out of the 5,334 cases. After excluding 118 cases (due to comorbid conditions listed in Fig. 1), 253 cases (213 ketosis without acidosis and 40 with DKA) were included in the data analysis. We randomly selected 221 cases without anaemia from the remaining 4,963 as the control group. Comorbid conditions of the control subjects are listed in Fig. 4. Within the entire study sample of 474 subjects, 129 had newly diagnosed T2DM and the remaining 345 had an established diagnosis of T2DM.

### Definitions and diagnostic criteria

The diagnosis of T2DM was established based on the 2014 ADA guidelines^2^. The diagnosis of ketosis was based on positive serum ketones (serum β-hydroxybutyrate level at >0.3 mmol/l) or moderate to large urine ketones (≥4 ml/L(2+))^34^. Ten patients with hypertonicity, ketosis and acidosis^46^,^47^ were also included in the ketosis group. Both blood ketones and urine ketones were required to be negative for inclusion as a control subject.

### Differential diagnosis

T1DM was identified by known history, uninterrupted insulin treatment, positive beta-cell autoantibodies and undetectable/low levels of plasma C-peptide during oral glucose tolerance test^2^. Two patients with maturity-onset diabetes of the young (MODY) were excluded on the basis of a prior diagnosis. One case of gestational diabetes mellitus (GDM) was also excluded from data analysis. Hyperglycemic hyperosmolar state (HHS) was identified based on the ADA criteria^34^. Anemia, caner, pancreatitis, end-stage chronic kidney disease, and hepatitis B were identified through clinical history, laboratory test results, or imaging studies. Starvation ketosis and alcoholic ketosis were distinguished by a history of chronic starvation or alcoholism and low plasma glucose concentrations or hypoglycemia^48^.

### Laboratory measurements and anthropometric index

All anthropometric and laboratory measures were obtained upon admission. The anthropometric information included age, gender, BMI, SBP, DBP, HR, and history of T2DM. Laboratory results included serum ketones (β-hydroxybutyrate), urine ketones (acetoacetic acid and acetone), Hb, HbA_1c_, PG, insulin, C-peptide, liver function tests (ALT and AST), renal function tests (sCr and BUN), UA, CRP, lipid profile (TG, TC, LDL, HDL, and FFA), arterial pH, base excess (BE), bicarbonate, electrolyte levels, and osmolality (Table 1). Serum ketones were measured using a MediSense hand-held device (Abbott Corporation, Abbott Park, IL, USA). Urine ketones were measured using the nitroprusside method (Semi-automatic urine analyzer, Cobas u411, Roche, Germany). HbA_1c_ was measured by high performance liquid chromatography (HLC-723G8, Tosoh, Japan). OGTTs were conducted prior to any treatment in all but 40 patients with diabetic ketoacidosis (after correction of acidosis). Insulin and C-peptide were measured during the OGTT. The formula, [*sodium* (*mEq*/*l*) × 2 + *glucose* (*mg*/*dl*)/18]^34^,^49^, was used to calculate effective osmolality.

### Multivariable model

The adjusted variables included in Model 1 were HbA_1c_, age, gender, and CRP. The variables included in Model 2 were HbA_1c_, age, gender, CRP, BMI, smoking, drinking, and diabetes duration. PG, insulin, and C-peptide levels were not included as independent variables due to the strong correlation with HbA_1c._ HbA_1c_ data were further divided into two factions depending on the HbA_1c_ threshold and was included in the multivariate models as a binary variable.

### Statistical methods

Continuous variables are presented as mean and standard deviation (SD) upon normal distribution, and as medians and interquartile ranges upon skew distribution. Categorical data are presented as percentages. Comparison of continuous variables between the cases and controls were conducted using Student’s *t*-test upon equal variance between the two groups, and otherwise using Mann-Whitney U test. Comparison of categorical variables between the cases and controls was conducted using a Chi-squared test (χ^2^-test). Rank correlations between HbA_1c_ and blood or urine ketones were determined using Spearman correlation coefficients. A step-wise binary logistic regression analysis was conducted to explore the risk factors for type 2 diabetic ketosis. ROC analysis was conducted to examine the sensitivity and specificity of HbA_1c_ and to determine the optimal HbA_1c_ threshold in order to discriminating ketosis from the entire sample. All statistical analyses were carried out using SPSS17.0 software (SPSS Inc., Chicago, IL, USA). Results were considered to be statistically significant at two-tailed *P* values less than 0.05.

## Additional Information

**How to cite this article**: Zhu, B. *et al*. HbA_1c_ as a Screening tool for Ketosis in Patients with Type 2 Diabetes Mellitus. *Sci. Rep.* **6**, 39687; doi: 10.1038/srep39687 (2016).

**Publisher's note:** Springer Nature remains neutral with regard to jurisdictional claims in published maps and institutional affiliations.

## Supplementary Material

Supplemental Table S1 and S2

## Figures and Tables

**Table 1:** Clinical Characteristics of Patients in the Ketosis and Control groups.

Characterizes	All (N = 474)	Type 2 diabetes (N = 221)	Type 2 diabetes + Ketosis (N = 253)	P-Value
Age (years)	52.8 ± 17.5	55.0 ± 16.6	50.9 ± 18.1	0.010^a^
Gender (Male)	310 (65.4%)	141 (63.8%)	169 (66.8%)	0.494
Diabetes history (year)				
0	129 (27.2%)	40 (18.1%)	89 (35.2%)	—
1~10	218 (46.0%)	113 (51.1%)	105 (41.5%)	—
10+	127 (26.8%)	68 (30.8%)	59 (23.3%)	—
BMI (kg/m2)	25.1 ± 4.5	25.3 ± 4.5	25.0 ± 4.6	0.542
Plasma ketones (mmol/l)	0.2 (0.0, 1, 8)	0.0 (0.0, 0.1)	1.7 (0.9, 3.4)	<0.001^a^
Urine ketones (ml/l)				
0 (0.8)	226 (47.7%)	221 (100%)	5 (2.0%)	
1+ (1.5)	25 (5.3%)	0	25 (9.9%)	
2+ (4.0)	65 (13.7%)	0	65 (25.7%)	
3+ (>8.0)	63 (13.3%)	0	63 (24.9%)	
4+ (>8.0)	95 (20%)	0	95 (37.5%)	
HbA1c (%)	10.1 ± 2.7	8.5 ± 2.0	11.5 ± 2.4	<0.001^a^
Hemoglobin (g/l)				
Male	143.4 ± 12.2	143.9 ± 9.8	142.9 ± 13.8	0.520
Female	129.9 ± 12.5	130.3 ± 7.0	129.5 ± 16.1	0.712
Admission glucose (mmol/l)	16.5 ± 7.7	12.0 ± 4.9	20.1 ± 7.7	<0.001^a^
FPG (mmol/l)	9.7 ± 4.0	8.2 ± 3.1	11.0 ± 4.2	<0.001^a^
2h-PG (mmol/l)	18.4 ± 5.3	17.0 ± 4.9	19.9 ± 5.4	<0.001^a^
Fasting insulin (pmol/l)	10.0 (6.0, 17.1)	10.3 (6.2, 18.1)	10.0 (5.8, 16.8)	0.180
2h-postprandial insulin (pmol/l)	22.8 (13.2, 38.2)	30.6 (17.6, 53.8)	17.2 (10.2, 29.3)	<0.001^a^
Fasting C-peptide (nmol/l)	1.7 (1.0, 2.3)	1.9 (1.3, 2.7)	1.4 (0.8, 2.0)	<0.001^a^
2h-postprandialC-peptide (nmol/l)	3.4 (2.3, 5.4)	4.7 (3.1, 6.9)	2.6 (1.8, 3.9)	<0.001^a^
TC (mmol/l)	4.8 ± 1.4	4.6 ± 1.1	4.9 ± 1.5	0.006^a^
TG (mmol/l)	1.4 (1.0, 2.3)	1.4 (1.0, 2.1)	1.4 (1.0, 2.5)	0.016^a^
LDL (mmol/l)	2.8 ± 1.0	2.7 ± 0.9	2.8 ± 1.1	0.372
HDL (mmol/l)	1.1 ± 0.3	1.1 ± 0.3	1.1 ± 0.4	0.713
FFA (mmol/l)	0.6 ± 0.3	0.5 ± 0.2	0.6 ± 0.3	<0.001^a^
Arterial PH	7.4 ± 0.1	7.4 ± 0.1	7.4 ± 0.1	0.057
Bicarbonate (mmol/l)	22.3 (17.8, 24.8)	25.5 (23.6, 26.4)	22.3 (17.8, 24.7)	0.232
BE (mmol/l)	−1.4 (−5.8, 0.7)	1.4 (−0.1, 2.7)	−1.6 (−6.0, 0.6)	0.005^a^
Osmolality (mOsm/kg)	296.6 ± 10.6	296.5 ± 6.4	296.6 ± 13.0	0.859
sCr (umol/l)	68.04 ± 28.60	70.4 ± 29.6	66.0 ± 27.6	0.097
BUN (mmol/l)	6.05 ± 2.56	6.1 ± 2.2	6.0 ± 2.9	0.916
AST (U/L)	27.1 (23.8, 30.4)	23.6 (20.6, 26.8)	30.1 (24.5, 35.7)	0.197
ALT (U/L)	33.4 (27.4, 39.5)	32.6 (21.6, 43.5)	34.7 (28.1, 41.2)	0.301

Continuous normal distribution variables are presented as means ± standard deviation (SD); continuous skew distribution variables are presented as medians (interquartile ranges); categorical data are given as numbers in percentage. BMI: body mass index; FPG: fasting plasma glucose; 2h-PG: 2 hours postprandial plasma glucose; TC: total cholesterol; TG: triglycerides; LDL: low density lipoprotein; HDL: high density lipoprotein; FFA: free fatty acids; BE: base excess; sCr: serum creatinine; BUN: blood urine nitrogen; AST: glutamic-oxalacetic transaminase; ALT: glutamic-pyruvic transaminase. ^a^*P* < 0.05.

**Table 2:** Parameters of the multiple logistic regression model.

Variables	βc	SE	Wald	P	OR	95% CI
Model 1						
HbA1c	0.65	0.07	91.32	0.001	1.87	(1.64–2.13)
Age	−0.01	0.01	1.44	0.231	0.99	(0.98–1.01)
Gender	0.05	0.27	0.03	0.866	1.05	(0.61–1.79)
CRP	0.04	0.01	16.83	0.001	1.04	(1.02, 1.05)
Model 2						
HbA1c	0.63	0.07	85.03	0.001	1.88	(1.64, 2.15)
Age	−0.01	0.01	0.97	0.325	0.99	(0.97, 1.01)
Gender	0.11	0.31	0.14	0.708	1.12	(0.62, 2.04)
CRP	0.04	0.01	15.02	0.001	1.04	(1.02, 1.06)

Adjusted variables in model 1: HbA1c, age, gender and CRP. Adjusted variables in model 2: HbA1c, age, gender, BMI, smoking, drinking, CRP and diabetes duration. βc: Regression coefficient. OR: odds ratio.

**Table 3:** Sensitivity, specificity, positive predictive value, negative predictive value, positive likelihood ratio, negative likelihood ratio and Youden index comparing various thresholds of HbA1c with the ADA criteria for diabetic ketosis in the subgroup of patients with previously diagnosed T2DM (n = 345).

HbA1c Thresholds	Sensitivity (%)	Specificity (%)	PPV (%)	NPV (%)	+LR	−LR	Youden index
8.3% (67 mmol/mol)	89.63 (83.94, 93.51)	57.00 (49.62, 63.90)	65.33 (58.90, 71.25)	85.83 (78.39, 91.06)	2.08 (1.75, 2.48)	0.18 (0.11, 0.29)	0.466
8.4% (68 mmol/mol)	88.41 (82.54, 92.53)	58.00 (50.73, 64.96)	65.61 (59.12, 71.56)	84.68 (77.22, 90.05)	2.11 (1.76, 2.52)	0.20 (0.13, 0.31)	0.464
8.5% (69 mmol/mol)	87.80 (81.84, 92.04)	60.00 (52.95, 67.07)	66.67 (60.13, 72.62)	84.50 (77.19, 89.81)	2.21 (1.83, 2.66)	0.20 (0.13, 0.31)	0.478
8.6% (70 mmol/mol)	86.59 (80.46, 91.04)	62.00 (54.62, 68.64)	67.30 (60.70, 73.28)	83.58 (76.32, 88.97)	2.27 (1.87, 2.76)	0.22 (0.15, 0.33)	0.486
8.7% (72 mmol/mol)	84.76 (78.41, 89.51)	62.00 (55.18, 69.16)	67.15 (60.48, 73.19)	81.88 (74.57, 87.47)	2.26 (1.85, 2.75)	0.24 (0.17, 0.36)	0.468
8.8% (73 mmol/mol)	81.10 (74.38, 86.39)	62.00 (55.18, 69.16)	67.17 (60.35, 73.34)	78.91 (71.58, 84.77)	2.26 (1.83, 2.78)	0.30 (0.21, 0.41)	0.431
8.9% (74 mmol/mol)	79.88 (73.05, 85.33)	66.00 (58.56, 72.28)	67.88 (60.99, 74.07)	78.29 (71.05, 84.14)	2.33 (1.88, 2.89)	0.31 (0.22, 0.42)	0.459

Values in parentheses are 95% confidence intervals.

PG: admission plasma glucose. PPV: positive predictive value. NPV: negative predictive value. +LR: positive likelihood ratio. −LR: negative likelihood ratio.

**Table 4:** Sensitivity, specificity, positive predictive value, negative predictive value, positive likelihood ratio, negative likelihood ratio and Youden index comparing various thresholds of HbA1c with the ADA criteria for diabetic ketosis in the subgroup of patients with newly diagnosed T2DM (n = 129).

HbA1c Thresholds	Sensitivity (%)	Specificity (%)	PPV (%)	NPV (%)	+LR	−LR	Youdenindex
9.7% (83 mmol/mol)	91.01 (83.03, 95.6)	55.00 (39.82, 69.3)	81.82 (72.99, 88.27)	73.33 (55.35, 86.02)	2.02 (1.43, 2.87)	0.16 (0.08, 0.34)	0.460
9.8% (84 mmol/mol)	89.89 (81.68, 94.79)	55.00 (39.82, 69.3)	81.63 (72.74, 88.14)	70.97 (53.25, 84.06)	2.00 (1.41, 2.83)	0.18 (0.09, 0.36)	0.449
9.9% (85 mmol/mol)	89.89 (81.68, 94.79)	60.00 (44.57, 73.68)	83.33 (74.52, 89.58)	72.73 (55.61, 85.1)	2.25 (1.53, 3.31)	0.17 (0.00, 0.33)	0.499
10.0% (86 mmol/mol)	89.89 (81.68, 94.79)	63.00 (47.00, 75.81)	84.21 (75.46, 90.31)	73.53 (56.71, 85.58)	3.00 (1.60, 3.60)	0.16 (0.08, 0.31)	0.529
10.1% (87 mmol/mol)	88.76 (80.36, 93.96)	65.00 (49.45, 77.92)	84.95 (76.18, 90.94)	72.22 (55.86, 84.30)	2.54 (1.65, 3.89)	0.17 (0.09, 0.32)	0.538
10.2% (88 mmol/mol)	86.52 (77.74, 92.27)	65.00 (49.45, 77.92)	84.62 (75.69, 90.73)	68.42 (52.45, 81.01)	2.47 (1.61, 3.80)	0.21 (0.12, 0.37)	0.515
10.3% (89 mmol/mol)	84.27 (75.19, 90.52)	65.00 (49.45, 77.92)	84.27 (75.19, 90.52)	65.00 (49.45, 77.92)	2.41 (1.56, 3.71)	0.24 (0.14, 0.41)	0.493

Values in parentheses are 95% confidence intervals.

PG:admission plasma glucose. PPV:positive predictive value. NPV:negative predictive value. +LR: positive likelihood ratio. −LR: negative likelihood ratio.
