# Supplementary material for: HbA1c as a Screening tool for Ketosis in Patients with Type 2 Diabetes Mellitus
Source: Sci Rep. 2016 Dec 23;6:39687. doi: 10.1038/srep39687 (PMC5180185; doi:10.1038/srep39687)
Supplement: Supplemental Table S1 and S2 [file srep39687-s1.pdf]

HbA1c as a Screening tool for Ketosis in Patients with Type 2 Diabetes Mellitus

Authors

Bing Zhu[1], Le Bu[1], Manna Zhang[1], Aaron M Gusdon[4], Liang Zheng[2], Sharvan Rampersad[1],

Jue Li[2*] & Shen Qu1,[3*]

Affiliations

[1]Department of Endocrinology and Metabolism, Shanghai Tenth People's Hospital, School of Medicine, Tongji University, Shanghai, China.

[2]Department of Epidemiology, School of Medicine, Tongji Universisy, Shanghai, China.

[3]Department of Endocrinology, School of Medicine, Nanjing Medical University, Nanjing, China.

[4]Department of Neurology and Neuroscience, Weill Cornell Medical College, New York-Presbyterian Hospital, New York, United States of America

[*]Co-corresponding authors:

E-mail: jueli@tongji.edu.cn (JL); qushencn@hotmail.com (SQ)

Supplemental Table S1-Laboratory findings in diabetic ketosis patients comparing patients with newly diagnosed T2DM and those with previously diagnosed T2DM.

Characterizes	Newly Diagnosed Type 2 DM (N=89)	Previously Diagnosed Type 2 DM (N=164)	P-Value
Age(years)	43.7±17.1	54.8±17.5	<0.001[a]
Gender(Male)	70(78.7%)	99(60.4%)	0.003[a]
Arterial PH	7.4±0.1	7.4±0.1	0.829
Bicarbonate (mmol/l)	22.5(19.5, 24.5)	22.7(18.0, 25.0)	0.392
BE (mmol/l)	-1.7(-6.0, 0.3)	-1.4(-5.9, 0.8)	0.586
K(mmol/l)	3.8±0.5	3.8±0.5	0.738
Na(mmol/l)	139.1±5.3	138.0±5.8	0.166
Cl(mmol/l)	99.9±11.7	99.8±5.4	0.896
Ca(mmol/l)	2.2(2.1, 2.3)	2.2(2.1, 2.3)	0.307
P(mmol/l)	1.1±0.3	1.0±0.4	0.188
Mg(mmol/l)	0.8±0.1	0.8±0.1	0.259
HbA1c (%)	12.3±2.0	11.1±2.5	<0.001[a]
Admission glucose(mmol/l)	22.2±8.3	18.9±7.1	0.002[a]
FPG(mmol/l)	10.1±3.4	11.5±4.5	0.021[a]
2h-PG(mmol/l)	19.7±5.2	20.0±5.5	0.804
Fasting insulin (pmol/l)	10.0(6.5, 14.2)	9.9(5.6, 17.6)	0.411
2h-postprandial insulin(pmol/l)	16.4(12.9, 27.1)	17.7(8.9, 31.0)	0.566
Fasting C-peptide(nmol/l)	1.5(1.0, 2.0)	1.2(0.6, 2.0)	0.457
2h-postprandial C-peptide(nmol/l)	2.9(2.1, 4.2)	2.5(1.7, 3.5)	0.090
TC (mmol/l)	5.0±1.6	4.9±1.4	0.707
TG (mmol/l)	1.7(1.1, 3.0)	1.3(0.9, 2.2)	0.122
LDL(mmol/l)	2.8±1.1	2.8±1.1	0.771
HDL(mmol/l)	0.9±0.3	1.1±0.4	<0.001[a]
FFA(mmol/l)	0.6±0.2	0.6±0.3	0.401

Continuous normal distribution variables are presented as means ± standard deviation (SD); continuous skew distribution variables are presented as medians (interquartile ranges); categorical data are given as numbers in percentage. FPG: fasting plasma glucose; 2hPG: 2 hours postprandial plasma glucose; TC: total cholesterol; TG: triglycerides; LDL: low density lipoprotein; HDL: high density lipoprotein; FFA: free fatty acids; BE: base excess. [a]$P < 0.05$.

Supplemental Table S2-Parameters of multiple logistic regression model.

Variables	ßc	SE	Wald	P	OR	95%CI
**Model 1**						
**HbA1c(a_1)**	**0.58**	**0.08**	**57.68**	**0.001**	**1.79**	**(1.54,2.08)**
**Model 2**						
**HbA1c(a_1)**	**0.60**	**0.08**	**54.75**	**0.001**	**1.81**	**(1.55,2.12)**
**Model 1**						
**HbA1c(a_2)**	**2.31**	**0.32**	**53.53**	**0.001**	**10.06**	**(5.42,18.70)**
**Model 2**						
**HbA1c(a_2)**	**2.53**	**0.35**	**53.55**	**0.001**	**12.49**	**(6.35,24.56)**
**Model 1**						
**HbA1c(b_1)**	**0.79**	**0.16**	**25.14**	**0.001**	**2.21**	**(1.62,3.00)**
**Model 2**						
**HbA1c(b_1)**	**0.92**	**0.18**	**25.35**	**0.001**	**2.51**	**(1.75,3.58)**
**Model 1**						
**HbA1c(b_2)**	**3.22**	**0.62**	**27.17**	**0.001**	**25.08**	**(7.47,84.25)**
**Model 2**						
**HbA1c(b_2)**	**3.32**	**0.65**	**26.35**	**0.001**	**27.58**	**(7.77,97.88)**

Adjusted variables in model 1: HbA1c, gender, age and CRP.

Adjusted variables in model 2: HbA1c, gender, age, BMI, smoking, drinking and CRP.

a_1: HbA1c data were continuous variables in patients with previously diagnosed type 2 diabetes.

a_2: HbA1c data were divided into two factions depending on the HbA1c threshold of 8.6% in patients with previously diagnosed type 2 diabetes.

b_1: HbA1c data were continuous variables in patients with newly diagnosed type 2 diabetes.

b_2: HbA1c were divided into two factions depending on the HbA1c threshold of 10.1% in patients with newly diagnosed type 2 diabetes.

ßc: Regression coefficient.
